# A light-activated magnetic bead strategy utilized in spatio-temporal controllable exosomes isolation

**DOI:** 10.3389/fbioe.2022.1006374

**Published:** 2022-09-06

**Authors:** Chenhan Wang, Duoteng Zhang, Haiyan Yang, Liang Shi, Lin Li, Changmin Yu, Jifu Wei, Qiang Ding

**Affiliations:** ^1^ Jiangsu Breast Disease Center, The First Affiliated Hospital with Nanjing Medical University, Nanjing, China; ^2^ Key Laboratory of Flexible Electronics (KLOFE) and Institute of Advanced Materials (IAM), Nanjing Tech University (NanjingTech), Nanjing, China; ^3^ Department of Pharmacy, Jiangsu Cancer Hospital and Jiangsu Institute of Cancer Research and The Affiliated Cancer Hospital of Nanjing Medical University, Nanjing, China

**Keywords:** exosome, cancer, magnetic separation, light-activated release, liquid biopsy

## Abstract

Tumor-derived exosomes are considered as a key biomarker in the field of liquid biopsy. However, conventional separation techniques such as ultracentrifugation, co-precipitation and column chromatography cannot isolate samples with high throughput, while traditional immunomagnetic separation techniques, due to steric effect of magnetic beads, reducing sensitivity of exosomes optical detection. Herein, we provide a novel and simple nanoplatform for spatiotemporally controlling extraction and elution of exosomes *via* magnetic separation and light-activated cargo release. In this system, magnetic beads are co-modified by photoresponsive groups -nitrobenzyl group and aptamers that are compatible with CD63-a highly expressed exosomal surface-specific protein. Through exosomes extracted from cell model and nude mice xenograft tumor model morphological characterization and proteomic analysis, results showed that our novel magnetic bead system outperformed current ultracentrifugation in serum exosome extraction in terms of extraction time, yield, and proportion of populations with high CD63 expression. This strategy may be a powerful tool for exosome isolation in clinical liquid biopsies of cancer disease.

## Introduction

Liquid biopsy is an emerging adjunctive detection method, which uses biological information carried by body fluid components such as blood, urine, sweat, et al. (Willms et al., 2018; Wang C et al., 2020; Descamps et al., 2022; Markou et al., 2022). Exosomes, a type of small extracellular vesicles with a size of 30–150 nm, are well-documented to participate in tumor development, e.g., stimulating the growth of tumor cells, suppressing anti-tumor immunity, promoting tumor cell migration and metastasis, et al. (Liu et al., 2018; Hofmann et al., 2020; Liao and Li, 2020; Chen et al., 2022; He et al., 2022). They are appealing to liquid biopsy due to following advantages: *1*) possessing diverse tumor biological information, such as RNAs, DNAs, and proteins; *2*) secreted by and indicating the activity of living tumor cells; *3*) easy to preserve and identify *via* their surface markers (Gurunathan et al., 2019; Sung et al., 2020; Yu et al., 2021). In fact, in some tumors, such as prostate and breast cancer, exosome-based liquid biopsies have shown significant prognostic value (Ibsen et al., 2017; Guo et al., 2020; Shen et al., 2020; Puhka et al., 2022).

Currently, exosomes are mainly isolated through ultracentrifugation (UC) (Momen-Heravi et al., 2013; Wang S et al., 2020; Xie et al., 2020; Yang et al., 2020). UC is normally time- and labor-consuming, and depends on availability of instrument. Some emerging exosome isolation methods, such as size separation-based chromatography column, microfluidic, immunosorbent-based kit *etc*. (Momen-Heravi et al., 2013; Gurunathan et al., 2019; Shen et al., 2020). These methods are cumbersome in extraction procedures and are not suitable for simple high-throughput detection (Lu et al., 2022; Zhao et al., 2022; Zhu et al., 2022). To solve the issues of centrifugal method, some new separation methods have been developed, such as magnetic bead system based on immunological binding and magnetic separation. Notably, in order to elute the captured exosomes from magnetic beads, non-physiological conditions of extreme ion concentrations are commonly involved, which may cause irreversible damage to exosomes during isolation and subsequently result in false information (Gao et al., 2019; Choi et al., 2022). Moreover, exosome detection methods based on magnetic separation have obvious steric hindrance due to large particle sizes of magnetic beads and exosomes. Detection technologies using fluorescence or chemiluminescence will weaken fluorescence signals due to masking or quenching effect of magnetic beads (Yang et al., 2015). Therefore, the development of a new magnetic bead system for efficient and gentle separation of exosomes has become an urgent need in the field of liquid biopsy.

**FIGURE 1:**
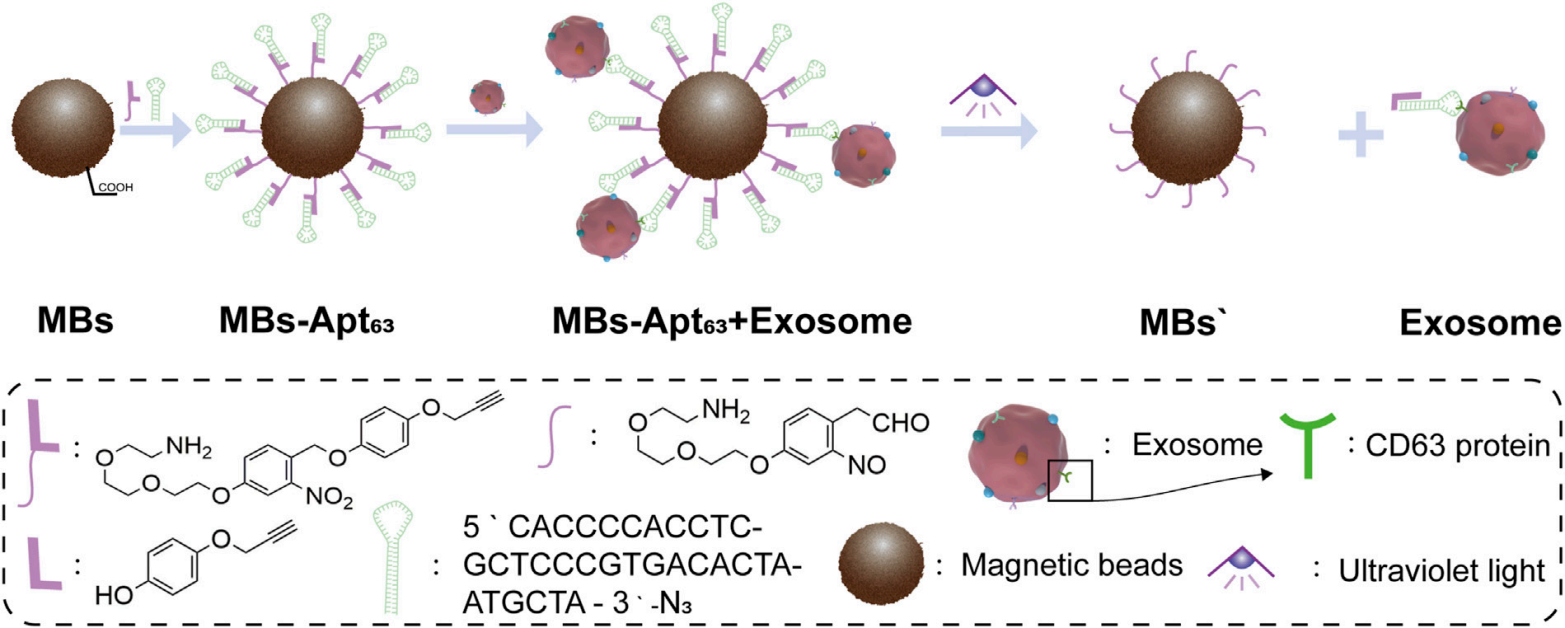
Schematic illustration of our ultraviolet-responsive nanomagnetic beads for binding and separation of exosomes.

Light is noninvasive, spatiotemporally controllable, and biocompatible, which is widely used as a trigger in clinical imaging diagnosis and treatment. This inspired us to exploit light-activated degradation to elude exosomes, which would allow spatial, temporal, and rate control by manipulating the location, timing, and intensity of applied light. Herein, we developed a novel strategy using magnetic bead-based capture and light-activated elution for spatiotemporally controllable exosome isolation from serum. As shown in Figure 1, we anchored CD63 aptamer on the surface of magnetic beads *via* light-sensitive nitrobenzene group, which was cleaved by ultraviolet (UV) light at around 365 nm (Chang et al., 2009; Kobayashi et al., 2012; Lai et al., 2016; Wang et al., 2017; Hentzen et al., 2020). CD63 is member of tetraspanins that is considered to be a reliable exosome surface marker. It plays important roles in membrane transport and has been proved to be a biomarker for breast cancer exosomes (Song et al., 2020; Mathieu et al., 2021). After incubation with serum from breast cancer-bearing mice, our magnetic bead system can selectively bind with the CD63 protein on exosomes, and subsequently isolate the target exosomes with external magnetic field. Finally, the structurally and functionally intact exosomes would be eluted from magnetic beads by regulating light excitation. This nanodevice provides a controllable strategy for separating exosomes from serum, allowing us to maximally preserve the value of exosomes for liquid biopsy and clinical studies. At the same time, the magnetic bead sorting method based on optical control strategy also provides a new platform for exosome detection of liquid homogeneous substances in the future.

## Materials and methods

### Materials and reagents

Magnetic beads (CSMN Beads-100, MBs, 100 nm) were purchased from So-Fe Biomedicine (Shanghai, China). The rest of chemical reagents used in the synthesis of nanodevices were purchased from Sinopharm Chemical Reagent Co. (Shanghai, China) and Sigma (Shanghai, China). SUM-1315 cells line was purchased from the Chinese Academy of Sciences (Shanghai, China). DMEM medium (11965092), Penicillin/streptomycin (15140122), and FBS (10099141C) were purchased from GIBCO (Shanghai, China). Anti-CD9 (13,403) primary antibodies were purchased from Cell Signaling Technology (Shanghai, China). Anti-CD63 (ab22595) and anti-calnexin (ab134045) primary antibodies were purchased from Abcam (Shanghai, China). Antirabbit HRP secondary antibodies (a0208), Enhanced-chemiluminescence (ECL) kits (P0018FS), and BCA kit (P0012S) were purchased from Beyotime. Biotechnology (Shanghai, China). CD63 aptamer with azide group was synthesized by Sangon (Shanghai, China), with the following sequence: 5′-CAC CCC ACC TCG CTC CCG TGA CAC TAA TGC TA-3′-N_3_ (Zhang Z et al., 2019).

### Synthesis of magnetic bead system (MBs-Apt_63_)

**FIGURE 2:**
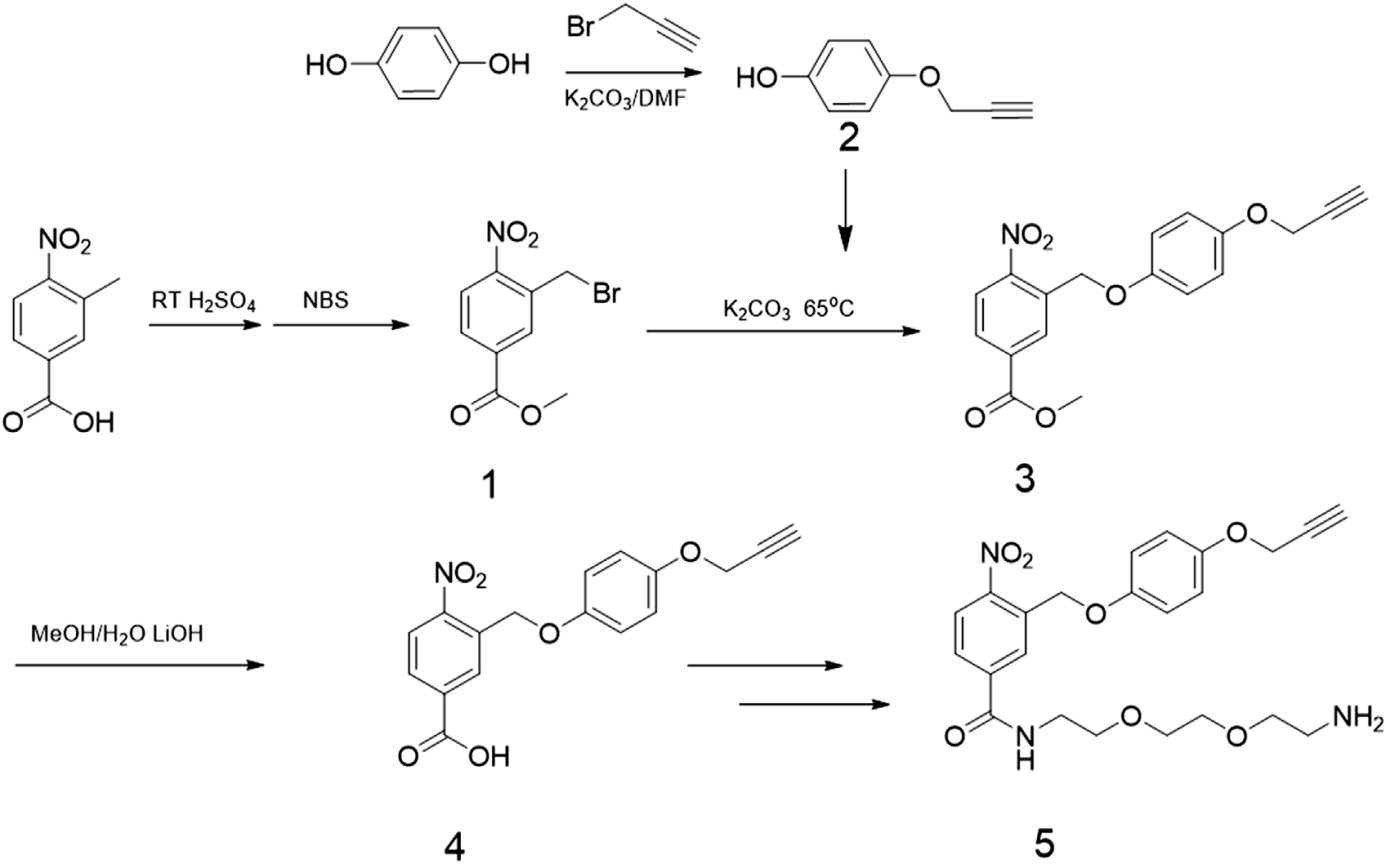
Synthesis routes of photo responsive ligands.

Magnetic nanoparticle solution (600 μl, 5 mg/ml) was added to a 1.5 ml centrifuge tube. The nanoparticles were precipitated to bottom of the centrifuge tube with magnets, and supernatant was removed. After being washed with water three times, the nanoparticles were redispersed in 3 ml of 2-morpholinoethanesulfonic acid (MES) buffer with ultrasound. 1-(3-Dimethylaminopropyl)-3-ethylcarbodiimide hydrochloride (EDC) (dissolved with MES, 2.5 mg/ml, 2 ml) and N-hydroxy succinimide (NHS) (dissolved with MES, 5 mg/ml, 1 ml) were then added, followed by sonication for 15 s. The centrifuge tube was sealed by sealing film and shaken at 37°C for 45 min. After that, the nanoparticles were deposited to the bottom of the centrifuge tube and washed twice with phosphate buffered saline (PBS) buffer. The nanoparticles were redispersed in 6 ml PBS. Compound 5 (the compound codes mentioned in this paper were shown in Figure 2) in dimethyl sulfoxide (DMSO) (60 μl, 100 mg/ml) was added to the centrifuge tube and mixed by ultrasound. The mixture was oscillated at 37°C for 12 h. After removal of the supernatant, the CD63 aptamer with azide group was added into the nanoparticles’ aqueous solution at a 1:1 M ratio. 1 mol% copper sulfate pentahydrate and 5 mol% sodium ascorbates were added and solution was shaken at room temperature for 8 h to get the final magnetic bead system (MBs-Apt_63_).

### Cell culture

SUM-1315 cells tested negative for *mycoplasma* were cultured in DMEM supplemented with 10% FBS and 1% penicillin−streptomycin. All cells were cultured in an incubator under 5% CO_2_ at 37°C.

### Agarose gel electrophoresis

The aptamer-MBs mixture system was loaded into each well of the agarose gel and separated *via* electrophoresis. Target oligonucleotides were detected with Gel-Red kits.

### Isolation of exosomes by UC from cell supernatant and serum

Exosomes were extracted from the supernatant of SUM-1315 cells or serum in the following steps: centrifugation was performed at 4°C at 2,000 × *g* for 10 min, and the supernatant was kept and centrifuged at 10,000 × *g* for another 30 min to further remove the debris. These samples were transferred to UC tubes and centrifuged at 110,000 × *g* for 75 min, followed by removal of the supernatant. The precipitates were resuspended and diluted with 1×PBS, and filtered through 0.22 µm membrane. The product was centrifugated again at 110,000 × *g* for 75 min, and the supernatant was discarded. The pellets were resuspended with 1×PBS and stored at −80°C for further use.

### Isolation of exosomes by MBs -Apt_63_

Magnetic beads (0.1 μg) were added to every 500 μl of exosome suspension or serum to capture exosomes. The binding process was carried out on a shaker. After 20 min, the EP tube was put in a tube rack with a permanent magnet placed against the tube bottom. The supernatant was discarded. After 5 min of precipitation, and the pellets were resuspended in PBS and placed under UV light for 20 min. After elution of the exosomes, the bare magnetic beads were removed by permanent magnets after of 5 min’s adsorption time, and the exosome-containing supernatant was retained in 500 μl PBS.

### Transmission electron microscope (TEM)

PBS solution (20 µl) containing exosomes was added to a carbon-coated copper grid and adsorbed. After cleaning with PBS, the copper grid was fixed in 1% glutaraldehyde for 2 min. The excess solution was removed by using paper towel to blot the edges of each grid. Then the sample was negatively stained with saturated uranyl acetate solution and incubated at room temperature for 1min. The unevaporated solution is removed with paper towel. The samples were observed under a transmission electron microscope.

### Nanoparticle tracking analysis (NTA)

Nanoparticle tracking analysis was performed on ZetaVIEW S/N 17-310 and all NTA measurements were performed using the same setup to ensure consistent results. The exosomes were diluted in PBS to obtain 50 particles in each field of vision for optimal counting.

### Dynamic light scattering (DLS)

Dynamic light scattering (DLS) measurements were conducted on a Nano ZS instrument (Malvern, United Kingdom). All samples were resuspended in PBS, and measured with the same instrument setup.

### Western blotting

Exosome suspension (10 μl) was loaded into SDS-PAGE and then separated by electrophoresis. Proteins were transferred onto polyvinylidene fluoride membranes and blocked with 5% skim milk. The primary anti-CD63 and anti-CD9 antibodies were used, followed by incubation with HRP-conjugated secondary antibodies. Target proteins were detected with enhanced-ECL kits.

### Animal models

BALB/C-nude mice (female, 4 weeks) were purchased from Model Animal Research Center, Nanjing University. The animal experiment was approved by the Ethics Committee of Nanjing Medical University. We confirmed that all animal experiments are complied with National Institutes of Health guide for the care and use of Laboratory animals (NIH Publications No. 8023, revised 1978). The animals were raised at 25°C with 60% humidity. 2 × 10^6^ of sum-1315 cells were inoculated into the back of each nude mouse. 10 days later, blood was collected from the posterior venous cluster of the eyeball. The blood was placed at room temperature for 2 h and centrifuged at 3000 rpm for 10 min at 4°C, and the upper serum was preserved. The serum of nude mice was stored at −80°C.

### High sensitivity flow cytometry

Exosomes were incubated with 20 µl fluorescently labeled antibody (CD63) at 37°C for 30 min in dark. Then 1 ml of pre-cooled PBS (4°C) was added, followed by centrifugation at 110,000 *g* for 70 min. The supernatant was carefully removed and 1 ml of pre-cooled PBS (4°C) was added to resuspend. The solution was then centrifuged at 110,000 *g* at 4°C for 70 min, and the supernatant was carefully removed, followed by resuspension of pellets in 50 µl of precooled PBS (4°C) for analysis.

### Proteomic analysis of exosomes

Exosome suspension was treated with 7 M urea, 2%SDS, and 1× Protease Inhibitor Cocktail. Protein concentration was determined using the BCA protein detection kit, and then the sample was processed with nuclease digestion, reduction/alkylation, acetone precipitation, and lysine-C/trypsin digestion. Desalination was carried out in the monospin column using 0.1% trifluoroacetic acid (TFA) and acetonitrile. After vacuum drying, 0.1%FA was added to redissolve the sample, and a 1–2 μg of sample was analyzed. The separation was performed with Easy-NLC 1000 (Thermo Scientific, United States) using an analytical column (C18, 1.9 μm, 75 μm × 20 cm) at a flow rate of 300 NL/min. Orbitrap Fusion Lumos (Thermo Scientific, United States) was used as a mass spectrometer. Data Dependent Acquisition (DDA) model was used for tandem mass spectrometry. The full-scan resolution was 60,000 (FWHM), the mass-charge ratio range was set to M/Z 350-1800, and the impact energy was set to 30% in HCD fragmentation mode. The original mass spectrometry data were collected and analyzed using the mass informatics platform Proteome Discoverer 2.4 (Thermo Fisher).

### Statistics

Origin 2019 and GraphPad Prism 8.0 were used for statistical calculation of all histograms, and one-way anova was used for calculation of mean and variance.

## Results

### Synthesis of UV-responsive magnetic bead system (MBs-Apt_63_)

The 2-nitrobenzyl groups were used as photo-cleavable linkers to anchor CD63 aptamers onto MBs. The detailed synthesis route is shown in Figure 2, and the chemical synthesis steps of photoresponsive ligands are described in Supplementary Material S1. The structure of MBs-Apt_63_ is shown in Figure 1. Firstly, the photo-cleavable linkers were anchored onto the surface of MBs *via* a moderate amination reaction. To selectively capture exosomes, CD63 aptamers were finally conjugated with the MBs through click reaction. The final UV-responsive magnetic bead system (MBs-Apt_63_) was characterized for Zeta potential, infrared spectroscopy, and dynamic light scattering (DLS) size (Supplementary Figure S2 and Supplementary Figure S10).

### Magnetic beads respond to UV *in vitro*

**FIGURE 3:**
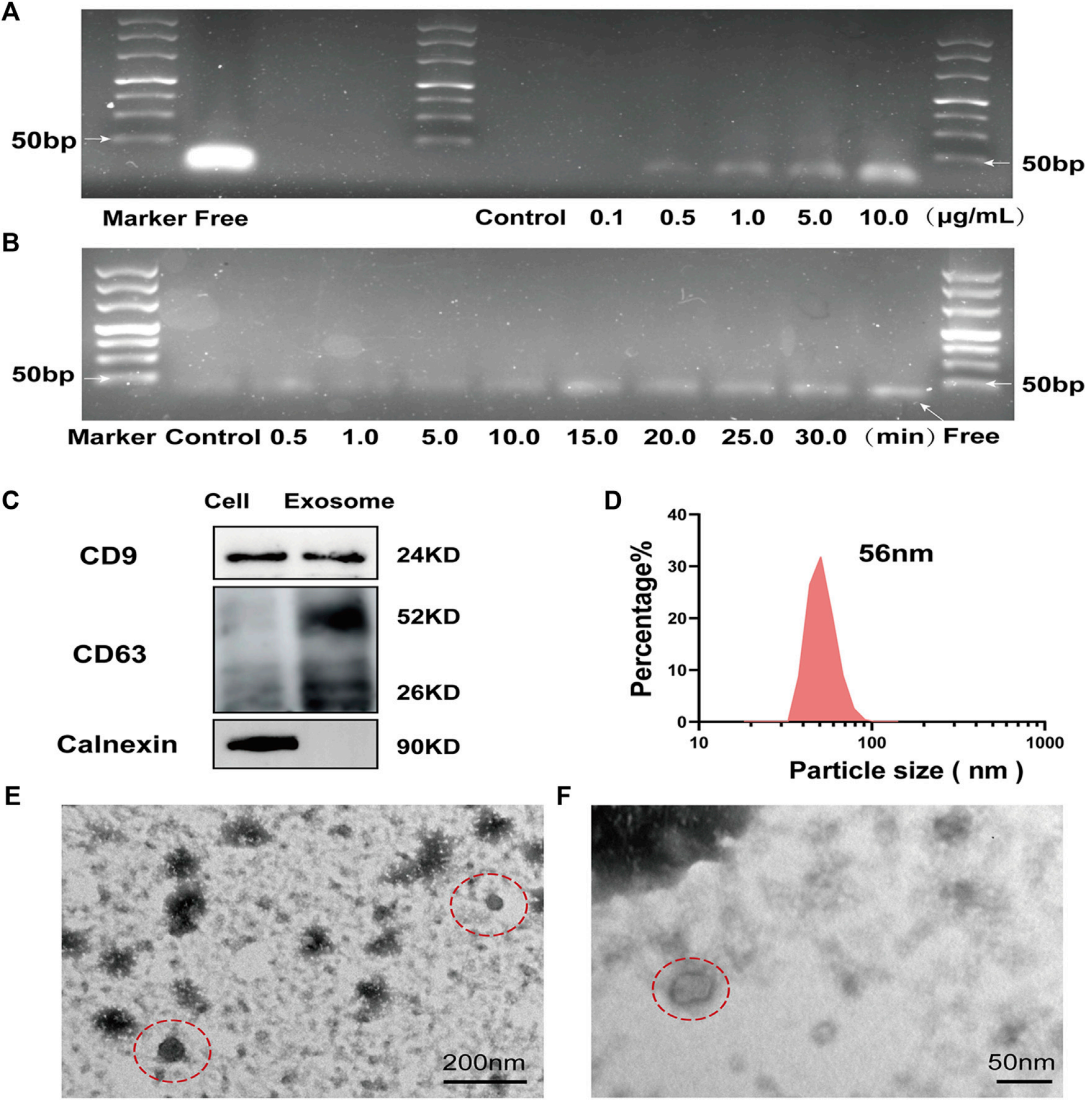
Response of MBs-Apt_63_ to UV *in vitro* and identification of exosomes **(A)** Agarose gel electrophoresis (molecular weight of free aptamer = 32 bp) of MBs-Apt_63_ at different concentrations after UV exposure; **(B)** Agarose gel electrophoresis of MBs-Apt_63_ (10.0 μg/ml) after exposure to UV for different times (molecular weight of free aptamer = 32 bp). **(C)** The expression of CD9, CD63 and calnexin determined by western blotting; **(D)** The particle size of breast cancer cell exosomes characterized by DLS; **(E**,**F)** Transmission electron microscopy image of breast cancer exosomes (magnification = 50000× in **C** and 100000× in **D**, exosomes are shown in the red dotted circle).

We measured the UV responsiveness of the magnetic beads with a concentration at the range of 0–10 μg/ml, while the time of exposure to UV light was between 0 and 30 min. In Figures 3A,B, it was found that within the range of 0–10 μg/ml, the light-responsive groups of magnetic beads were highly sensitive to and effectively cleaved by UV light. The CD63 aptamers were rapidly released from MBs-Apt_63_ under irradiation, which also showed obvious time dependence. With increased exposure time, the amount of released free aptamers increased, and reached the maximum after 15 min of UV exposure.

### Preparation and validation of exosomes

To test the ability of MBs-Apt_63_ to capture exosomes, we firstly extracted tumor exosomes from the cultural medium of breast cancer cell line SUM-1315 by UC. Western blotting analysis of exosomes showed the presence of CD9, CD63 (two common surface markers of exosomes) and calnexin (endoplasmic reticulum protein), indicating the successful preparation (Figure 3C). In DLS analysis, the exosomes showed an average size of 56 nm (Figure 3D), in line with characteristic particle size of exosomes (30–150 nm). Finally, we used TEM to observe the morphology of exosomes (Figures 3E,F), which displayed a typical lipid bilayer structure and a size in the range of 50–100 nm.

### Capturing exosomes with MBs-Apt_63_ and elution with UV light

**FIGURE 4:**
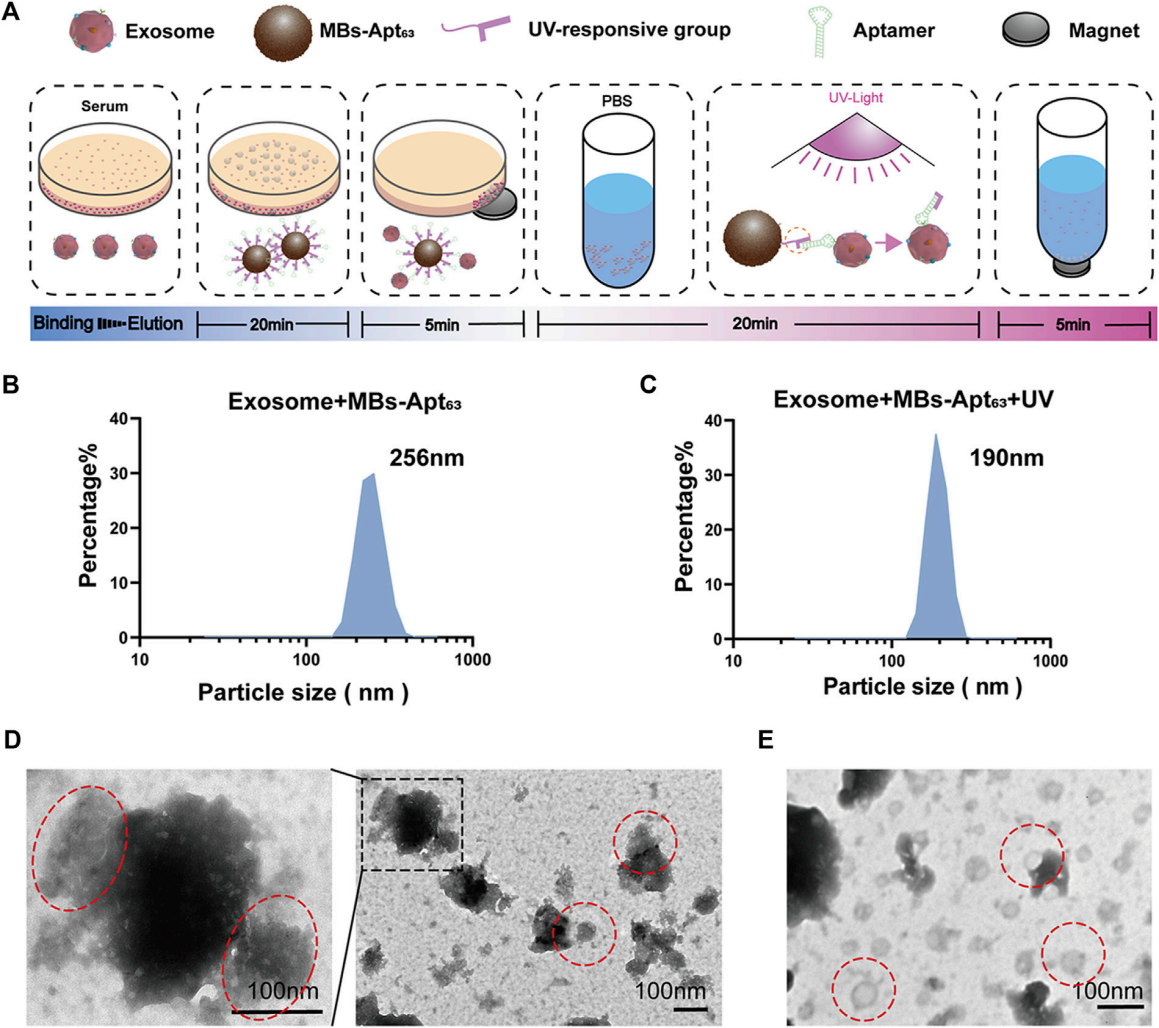
Capturing exosomes by and elution of exosomes from MBs-Apt_63_. **(A)** Schematic diagram for extracting exosomes from serum and cell supernatant with MBs-Apt_63_; **(B)** DLS characterization after incubating exosomes with MBs-Apt_63_; **(C)** DLS characterization after exposing the MBs-exosome complex to UV light; **(D)** Transmission electron microscopy image of exosomes bound to MBs-Apt_63_ (left, 100000×, right, 25000×). Positions of exosomes and MBs-Apt_63_ are marked in red; **(E)** Transmission electron microscopy image of exosomes eluted from MBs-Apt_63_ by UV light.

The process of isolating exosomes from cell supernatant or serum is shown in Figure 4A, which involves beads binding and exosome elution. DLS analysis was performed after MBs binding to exosomes and UV-triggered elution, which showed a size of 250 and 190 nm, respectively. (Figures 4B,C). In Figure 4C, the magnetic bead components were not removed in the system after ultraviolet dissociation. This reduced shift in maximum peak particle size from 256 to 190 nm indicated the elution of exosomes from the MBs after UV exposure. To further elaborate the binding and elution processes, TEM was used to observe the MBs-exosome complex before and after UV-triggered cargo release. Typical MBs bound to many exosomes were clearly seen before exposure to UV light (Figure 4D), whereas only purified exosomes could be observed after UV-induced release of exosomes and removal of magnetic beads by permanent magnets (Figure 4E).

### Application of MBs-Apt_63_ in the separation of serum exosomes from breast cancer

**FIGURE 5:**
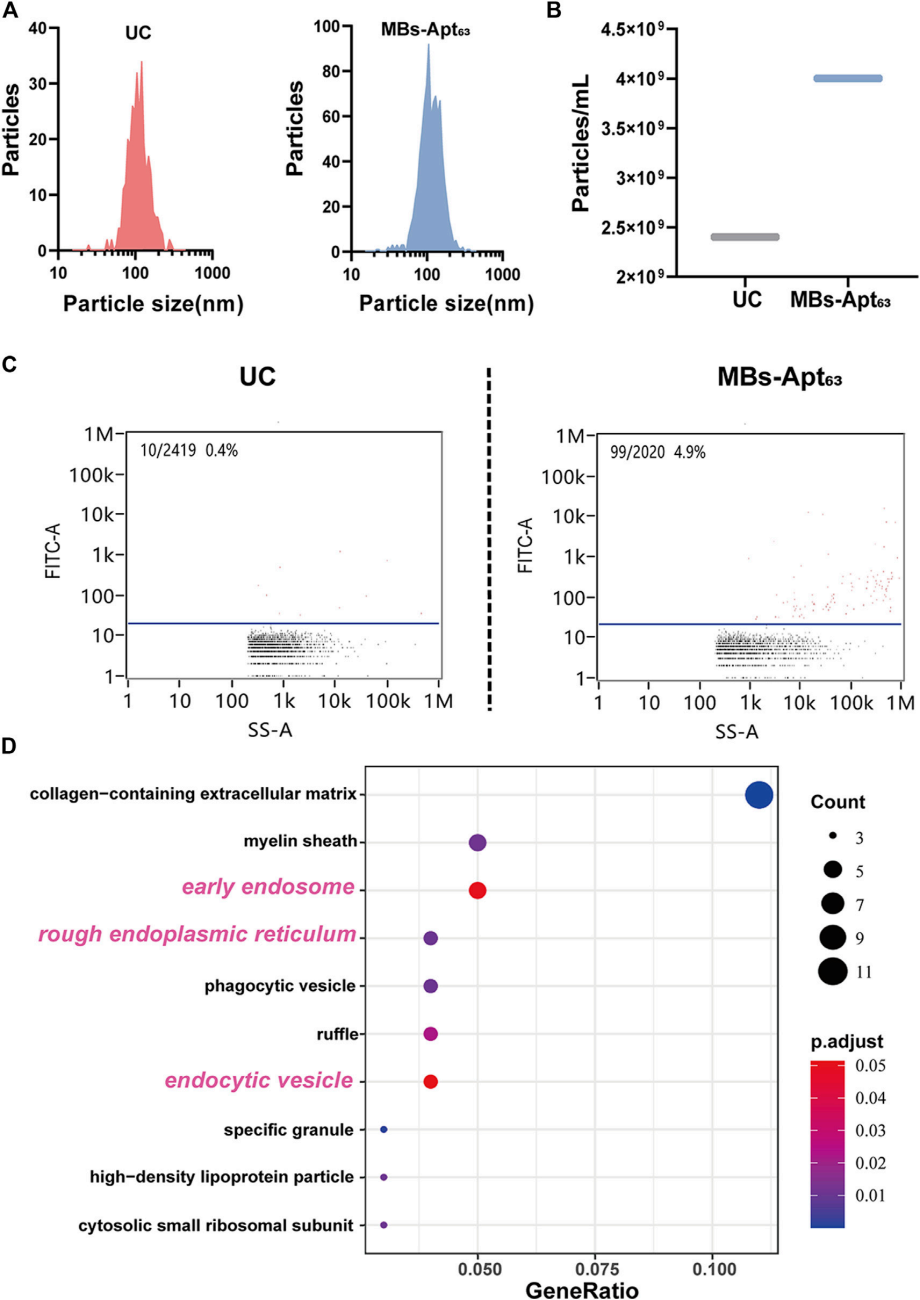
Evaluation of MBs-Apt_63_ in the isolation of serum tumor exosomes. **(A)** Particle size distributions of exosomes extracted by UC and MBs-Apt_63_. **(B)** Average serum exosome concentrations prepared by UC or MBs-Apt_63_; **(C)** Expression abundance of CD63 in exosomes isolated by UC and MBs-Apt_63_. Results were obtained from nanoflow flow test; **(D)** GO analysis of proteins from exosomes separated by MBs-Apt_63_ or UC. Top 10 GO components are displayed that are most enriched in exosomes separated by MBs-Apt_63_ (*p* < 0.05 as significance).

To evaluating the performance of this novel MBs system in isolating serum exosomes, we established mice models of triple-negative breast cancer using human breast cancer SUM-1315 cells. We collected the serum when the tumor grew to about 800 mm^3^, and then isolated exosomes with MBs-Apt_63_ and conventional UC respectively. We firstly analyzed the particle size distribution (Figure 5A) and particle concentration of exosomes (Figure 5B) with NTA. Results showed that the particle size distribution of exosomes isolated by MBs-Apt_63_ and UC was similarly uniform, both of which were about 100 nm. While the serum concentration of exosomes acquired by UC is lower compared with that by MBs-Apt_63_ (2.5 × 10^9^/ml versus 4.0 × 10^9^/ml) according to the particle count analysis, indicating higher efficiency of MBs-Apt_63_ in extracting serum exosomes.

Next, we used the high sensitivity flow cytometry (HSFC) system to analyze the expression of CD63 on exosomes separated by UC and MBs-Apt_63_ (Figure 5C). The results showed that the expression abundance of CD63 was 4.9% in exosomes extracted by MBs-Apt_63_, which is much higher (0.4%) in exosomes separated by UC method. Therefore, the light strategy in MBs-Apt_63_ system improved the purity of exosomes with high expression of CD63.

In exosome-based liquid biopsies, diagnostic value ultimately comes from biomarkers in exosomes. In this study, proteins in exosomes separated by UC and magnetic beads were both analyzed by liquid mass spectrometry. We conducted a database search and comparison according to Proteome Discoverer 2.4 system. Specific database search parameters are shown in Supplementary Table S2. The relevant quality control information is shown in Supplementary Figure S5. 1044 proteins were identified in the exosomes isolated by UC, while only 677 proteins were detected in the exosomes separated by magnetic beads (Supplementary Table S3). To verify that our optically controlled MBs-Apt_63_ preserved more exosome-related proteins, we performed GO analysis on high-expression set of exosome proteins extracted by MBs-Apt_63_ versus UC. The screening criteria for differential proteins between the two groups were:

(1), Inter-group ratio ≥ 4 (2), Unique Peptides ≥ 2.

Among the top 10 GO components that were significantly higher in exosomes extracted by MBs-Apt_63_, three are related to exosome formation, including rough endoplasmic reticulum, early endosomes, and endocytic vesicles (Figure 5D), indicating a higher expression abundance of exosome-related proteins in CD63 positive exosomes separated by MBs-Apt_63_.

## Discussions

In this study, we developed a facile light-responsive magnetic bead sorting system for extracting exosomes from breast cancer. In our strategy, 2-nitrobenzene is designed as the surface ligand of the magnetic beads, whose UV responsiveness allows for spatiotemporal control of exosome separation with minimum interference with serum composition. From a practical perspective, it requires less time and smaller sample volume, and does not need sophisticated instruments. More importantly, this system can largely improve the efficiency of exosome isolation, maximizing the purity and integrity of the exosomes. At the same time, without the interference of magnetic beads, exosomes in the real liquid environment can greatly improve the efficiency of the labeled enzyme and substrate reaction. In addition, the masking the effect of magnetic beads themselves will be eliminated. Therefore, this method should be able to improve the sensitivity of subsequent detection.

Previous studies have reported considerable progress in the field of liquid biopsy, particularly some nanomaterial-based liquid biopsy techniques (Zhang P et al., 2019; Tian et al., 2019; Huang et al., 2020; Xia et al., 2020; Chen et al., 2021; He et al., 2021). On this basis, some studies have significantly improved the sensitivity of liquid biopsies, while others have looked at reducing the amount of fluid used in this process (Huang et al., 2019; Li et al., 2019; Kim et al., 2020; Jiang et al., 2021). In this study, the operation mode and sample requirements of liquid biopsy were further simplified. Firstly, the extraction method of exosomes by illumination and magnetic separation does not require additional reagents and processing time, which ensures reproducibility of extraction procedures, making our magnetic bead system can be used in large quantities in clinical practice. Secondly, the spatiotemporally controllable extraction method provides a simple exosome release strategy to remove the steric effect based on magnetic bead fixation, so that magnetic bead-based exosome detection can achieve better detection efficiency in the liquid phase reaction system. At the same time, as most research on UV response devices, the wavelength of UV light used in this study is about 254 nm. It has been shown in some studies that there is no relevant evidence to confirm that 254 nm ultraviolet light will cause damage to proteins under certain conditions of time and intensity (O'Brien et al., 2014; Cannon et al., 2014).

Our experimental results basically demonstrate the clinical application prospect of the system. Compared with traditional ultracentrifugation, the number of exosomes extracted from serum by our system increased by 60%. At the same time, the purity of proteins represented by CD63 was increased by 10 times. Through proteomic analysis, we also confirmed that the MBs-Apt_63_ preserved more protein components from exosomes. It indicated that, compared with traditional exosome extraction methods in the field of liquid biopsy for clinical cancer patients, this system can obtain the information of tumor in patients’ serum in a relatively comprehensive way, thus providing more reference value for implementation of diagnosis and treatment process. In the future, by changing the aptamers, our strategy provides a flexible approach to pathological analysis of breast cancer for personalized medicine, and its large-scale application can also supply a new platform for broadening the application scope of liquid biopsy of cancer. At the same time, this light-activated magnetic bead strategy also establishes the possibility to nondestructively enrich specific functional cell exosomes for cancer disease treatment (Fang et al., 2022).

## Data Availability

The original contributions presented in the study are included in the article/Supplementary Material; further inquiries can be directed to the corresponding authors.
