# Supplementary material for: A light-activated magnetic bead strategy utilized in spatio-temporal controllable exosomes isolation
Source: Front Bioeng Biotechnol. 2022 Sep 6;10:1006374. doi: 10.3389/fbioe.2022.1006374 (PMC9486319; doi:10.3389/fbioe.2022.1006374)
Supplement: Supplementary file 1 [file DataSheet1.docx]

**Supporting information**

**A light-activated magnetic bead strategy utilized in spatio-temporal controllable exosomes isolation**

Chenhan Wang^1†^, Duoteng Zhang^2†^, Haiyan Yang^1†^, Liang Shi^1^, Lin Li^2^, Changmin Yu^2*^, Jifu Wei^3*^, Qiang Ding^1*^

^1^Jiangsu Breast Disease Center, the First Affiliated Hospital with Nanjing Medical University, Nanjing, 210029, P. R. China;

^2^Key Laboratory of Flexible Electronics (KLOFE) & Institute of Advanced Materials (IAM), Nanjing Tech University (NanjingTech), 30 South Puzhu Road, Nanjing, 211800, P. R. China;

^3^Department of Pharmacy, Jiangsu Cancer Hospital & Jiangsu Institute of Cancer Research & The Affiliated Cancer Hospital of Nanjing Medical University, Nanjing, 210018.P. R. China.

Chenhan Wang^1†^, Duoteng Zhang^2†^and Haiyan Yang^1†^these authors contributed equally to this work and share first authorship.

* Corresponding author

Qiang Ding^1*^

E-mail: [dingqiang@njmu.edu.cn](mailto:dingqiang@njmu.edu.cn)

Jifu Wei^3*^

E-mail: weijifu@njmu.edu.cn

Changmin Yu^2*^

E-mail: [iamcmyu@njtech.edu.cn](mailto:iamcmyu@njtech.edu.cn)

**1. Synthesis of photoresponsive ligands**

**Fig. S1** Synthesis routes of photoresponsive ligands (same as in **Fig.2**)

Compound **1**: 0.3 mL H_2_SO_4_ was slowly added to a flask with 20 mL methanol solution under agitation, then 3-methyl-4-nitrobenzoic acid (1 g, 5.5 mmol) was dissolved in a 20 mL methanol-sulfuric acid mixture and heated at reflux for 8 h. The reaction was monitored by TLC until the reaction stopped, and then the flask was cooled to room temperature. Neutralize in an aqueous solution of saturated sodium bicarbonate under ice-water bath conditions until no bubbles are formed in the solution. 20 mL water was added to the flask and extracted with ethyl acetate for 5 times. The organic layer was dried with anhydrous sodium sulfate and take 200 mg solid into a 50 mL round-bottom flask. Then the azodiisobutyronitrile (AIBN, 16.4 mg, 0.1 mmol) was dissolved in the round-bottom flask by adding 2 mL carbon tetrachloride (CCl_4_). Then N-bromosuccinimide (213.6 mg, 1.2 mmol) and CCl_4_ (1.5 mL) were added into a centrifuge tube and dropped into the round-bottom flask. After that, the round-bottom flask was placed in the oil bath and raised to 47°C for stirring for 1 h, then raised to 85°C for stirring 10 h. The reaction was monitored by TLC until the end of the reaction. The organic solvent was then extracted with dichloromethane (DCM) and saturated salt water and the organic phase was dried with anhydrous sodium sulfate. Crude product was purified by column chromatography. The pale yellow solid was compound **1** (223 mg) with a yield of 68%.

^1^H NMR(500 MHz, DMSO):δ 8.65 (s, 1 H), 8.23 (d, J = 8.0 Hz, 1H), 7.67 (d, J = 8.0 Hz, 1 H), 4.84 (s, 2 H), 3.97 (s, 3 H).

Compound **2**: Firstly, K_2_CO_3_ (3.73 g, 27 mmol) was added into a 500 mL round-bottomed flask, then hydroquinone (1 g, 9.0 mmol) was added to the flask. The solid was dissovled by 150 mL anhydrous acetone and stirred for 15 min at room temperature. Bromopropyne (1.06 g, 9.0 mmol) was added slowly to the mixture. The round-bottom flask was put into the oil bath and refluxed for 12 h. The reaction was monitored according to the TLC until the end of the reaction. Then the potassium carbonate K_2_CO_3_ was removed by suction filtration, and the organic solvent was vacuum-dried, washed with saturated salt water and extracted with EA. The compound **2** (746 mg) was a brown oily substance with a yield of 56%.

^1^H NMR (500 MHz, CDCl3) δ 6.86 (d, J = 9.0 Hz, 2 H), 6.77 (d, J = 9.0 Hz, 2 H), 4.62 (d, J = 2.4 Hz, 3 H), 2.50 (s, 1 H).

Compound **3**: K_2_CO_3_ (3.73 g, 27 mmol) and compound **2** (1.33 g, 9 mmol) were dissolved in 200 mL anhydrous acetone. Then compound **1** (3.01 g, 11 mmol) was added slowly to the mixed solution under agitation conditions. The round-bottomed flask was put into the oil bath and refluxed for 12 h. The reaction was monitored according to TLC until the end of the reaction, and then the K_2_CO_3_ was removed by suction filtration. The organic solvent was washed with saturated salt water and extracted with EA. After that, the organic phase was dried with anhydrous sodium sulfate and purified by rapid column chromatography. The compound **3** (2.15 g) was a pale yellow solid with a yield of 70%.

^1^H NMR (500 MHz, CDCl3) δ 8.53 (s, 1 H), 8.13 (s, 2 H), 6.94 (d, J = 4.4 Hz, 4 H), 5.38 (s, 2 H), 4.63 (d, J = 2.4 Hz, 2 H), 3.96 (s, 3 H), 2.51 (t, J = 2.4 Hz, 1 H). ^13^C NMR (126 MHz, CDCl3) δ 165.21, 152.80, 152.41, 149.42, 134.70, 134.18, 130.14, 129.52, 124.99, 116.08, 78.73, 77.33, 77.0, 76.82, 75.50, 67.16, 56.45, 52.90.

Compound **4**: The mixed solution (THF/MeOH/H_2_O = 4:1:1) in a certain proportion was added into 10 mL centrifuge tube. Lithium hydroxide (LiOH, 35.446 mg, 1.5 mmol) was added into the flask, and the mixed solution was slowly added under the condition of agitation. After the temperature of the mixed solution was reduced to room temperature, compound **3** (125 mg, 0.37 mmol) was added and stirred at room temperature for 1 h. The reaction was monitored according to TLC until the end of the reaction. The organic phase was dried with anhydrous sodium sulfate and rotated in vacuum to obtain light yellow solid (115 mg). The yield of compound **4** was 95% after the next reaction.

Compound **5**: Compounds **4** (200 mg, 0.61 mmol) and 1-(3-dimethylaminopropyl) -3-ethylcarbondiimide hydrochloride EDCI (233 mg, 1.22 mmol) were added into a 25 mL round-bottom flask, followed by 8 mL tetrahydrofuran (THF) solution. N-hydroxythiosuccinimide (NHS, 171.4 mg, 0.793 mmol) was added after the mixture was evenly stirred. After stirring at room temperature for 5 h, the reaction was monitored according to TLC. It was found that compound **5** was completely reacted, the reaction was stopped, and the THF solvent was removed. 8 mL of N, n-dimethylacrylamide (DMF) was added to fully dissolve solids in the round-bottom flask. 2,2- (ethane-1, 2-diyl bis (oxy)) bis (ethane-1-amine) (361.63 mg, 2.44 mmol) was added to the round-bottom flask, and stirred at room temperature for 6 h. The reaction was monitored according to TLC. The organic phase was dried with anhydrous sodium sulfate and purified by rapid column chromatography. After the drying, light yellow solid compound **5** was obtained (139 mg). The yield of compound 5 was 50%.

^1^H NMR (500 MHz, DMSO-d6) δ 9.14 (t, J = 5.5 Hz, 1 H), 8.28 (s, 1 H), 8.17 (d, J = 8.5 Hz, 1 H), 8.11 (s, 1 H), 6.99 – 6.89 (m, 4 H), 5.39 (s, 2 H), 4.71 (d, J = 2.3 Hz, 2 H), 3.61 (t, J = 5.4 Hz, 2 H), 3.56 (s, 6 H), 3.43 (s, 3 H), 2.89 (t, J = 5.4 Hz, 2 H), 1.22 (s, 2 H).

**2. Characterization of MBs-Apt63
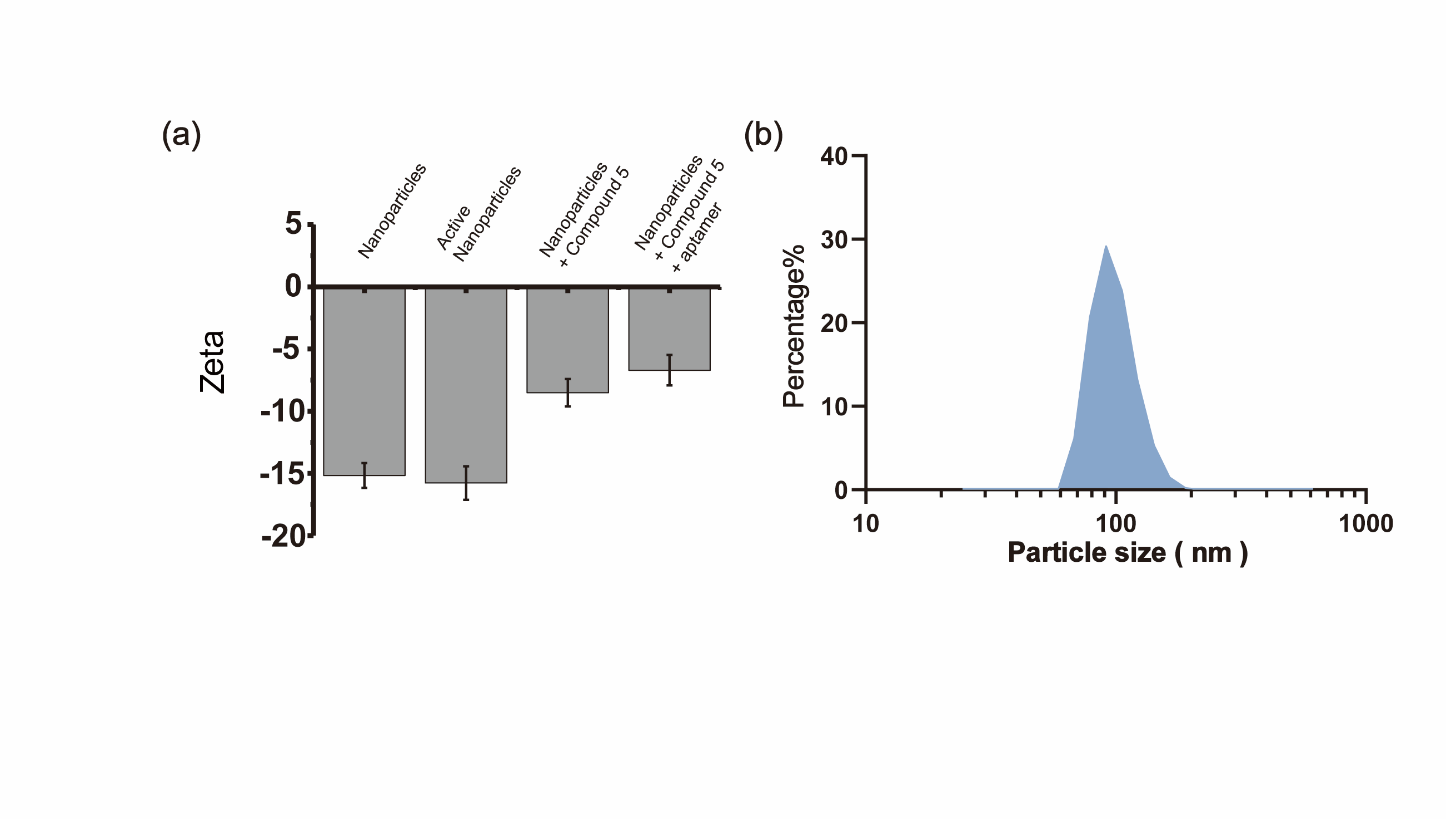
**

**Fig. S2 Potential characterization and DLS characterization of MBs-Apt63. (a)** Zeta potential changes of magnetic beads and MBs-Apt_63_;**(b)** DLS of MBs-Apt_63_.

1. **Blank control for nanoflow flow analysis of exosomes**

**
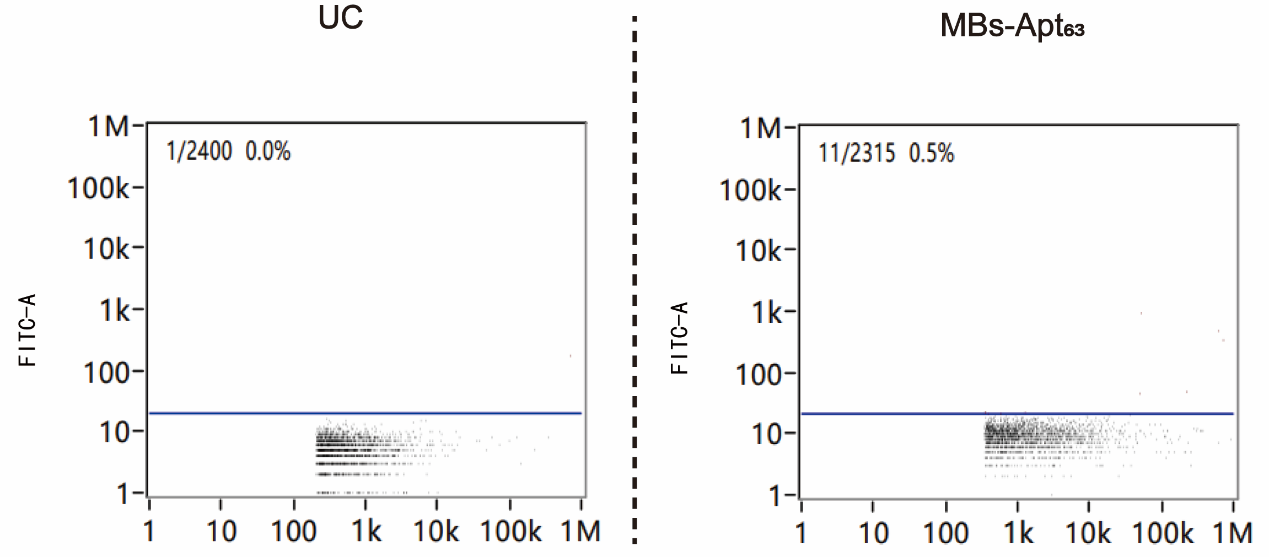
**

**Fig. S3 CD63 expression in the blank control group was determined by flow cytometry.** The quality control results of the UC group and MBs-Apt63 extraction group were analyzed under the same conditions.

**4. Quality control data for exosome protein analysis**


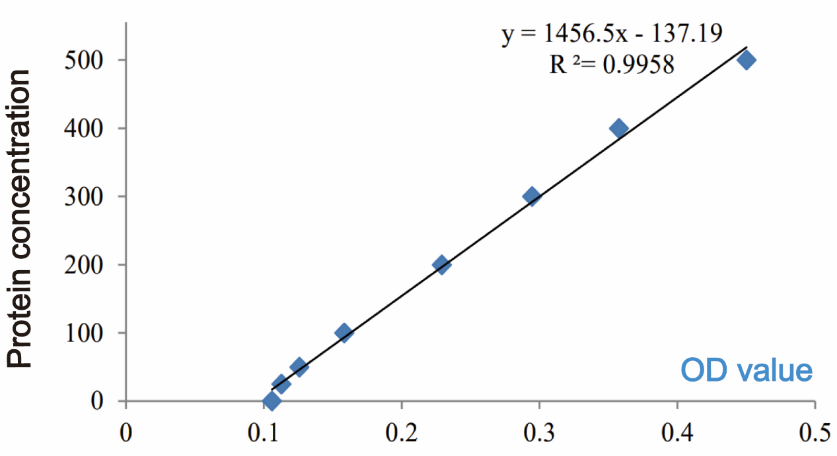


**Fig. S4 Standard curve for protein concentration determination by BCA method.** The final concentration of samples is shown in Table.S1.


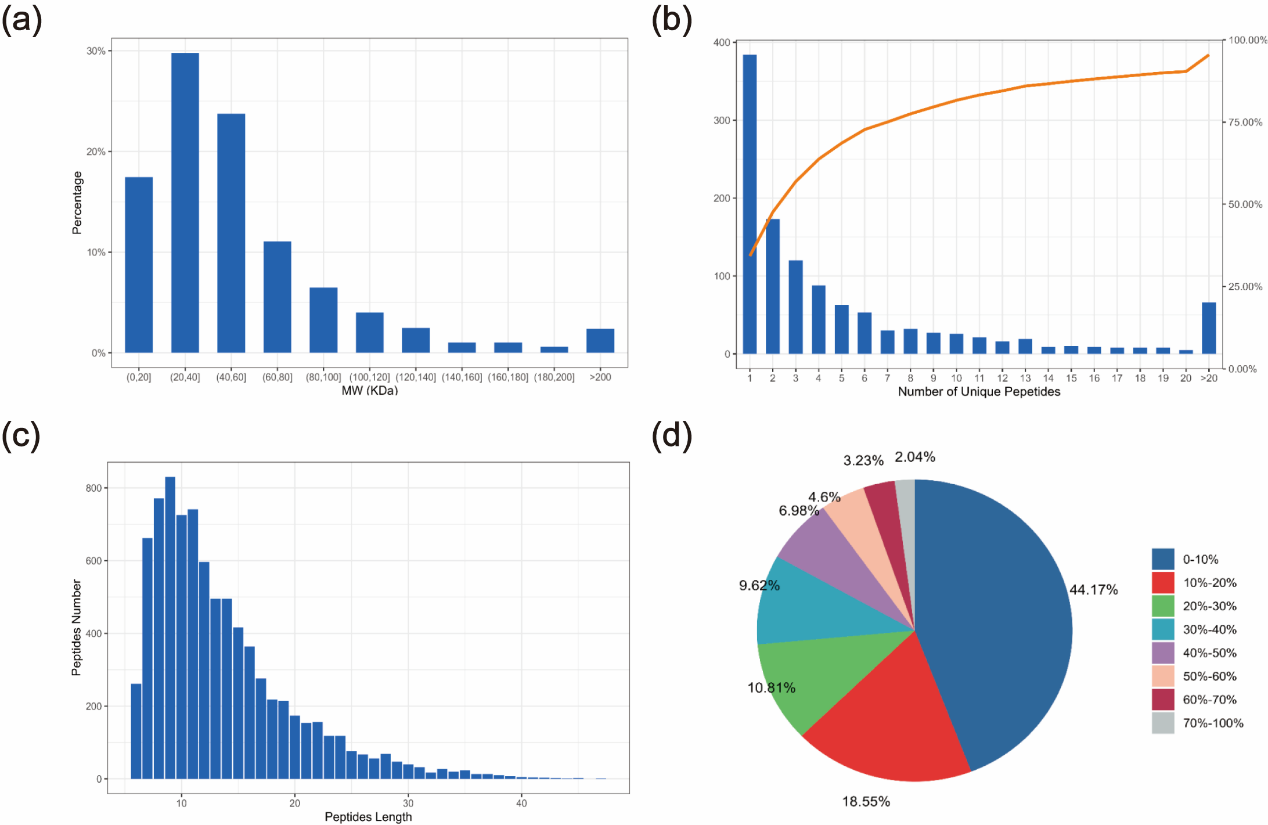


**Fig. S5 Relevant peptide and protein quality control information. (a)** Relative molecular weight distribution of proteins Abscissa: molecular weight of identified proteins, unit: thousand Dalton (kDa); ordinate: percentage of identified proteins; **(b)** Identify the distribution of unique peptide numbers in proteins. The left ordinate and abscissa indicate the number of proteins corresponding to different unique peptide numbers. The curve (right ordinate and abscissa) showed the proportion of the protein number corresponding to the total number of unique peptides to the total number of proteins; **(c)** Peptide sequence length distribution, the horizontal coordinate is the peptide length (that is, the number of amino acids contained in the peptide), the vertical coordinate is the total number of peptide corresponding length; **(d)** Protein identification coverage distribution Pie chart: Protein identification (95% confidence peptide) coverage distribution The pie charts with different colors represent the percentage of proteins with different identification coverage ranges.

**Table. S1 Determination of exosome protein concentration by BCA protein method.**

| Sample | Group | Concentration (μg/μL) |
| --- | --- | --- |
| Exosome | UC | 0.55 |
| Exosome | MBs-Apt_63_ | 3.16 |

**Table. S2 Proteome Discoverer 2.4 Database search parameters set in the system.**

| Item | Parameter values |
| --- | --- |
| Database | Swissprot |
| Taxonomy Mus | Musculus |
| Enzyme | Trypsin |
| Fixed modifications | Carbamidomethyl (C) |
| Variable modifications | Oxidation (M),Acetyl (Protein N-term) |
| Max Missed Cleavages | 2 |
| Target FDR (Strict)  Target FDR (Relaxed) | 0.01  0.05 |
| Min. peptide Length | 6 |
| Mass Tolerance | 10 ppm |

**Table. S3 Statistical results of protein and peptide in samples.**

| Item | Parameter values |
| --- | --- |
| Total protein number | 1175 |
| Total peptides number | 8320 |
| Unique peptides number | 7518 |
| Number of PSMs | 20270 |
| Number of proteins corresponding to UC group | 1044 |
| Number of proteins corresponding to MBs-Apt_63_ group | 677 |

**5. ^1^H NMR, ^13^C NMR and IR spectra**

**Fig. S6. ^1^H NMR spectrum of compound 1.**

**Fig. S7. ^1^H NMR spectrum of compound 3.**

**Fig. S8. ^13^C NMR spectrum of compound 3.**

**Fig. S9. ^1^H NMR spectrum of compound 5.**


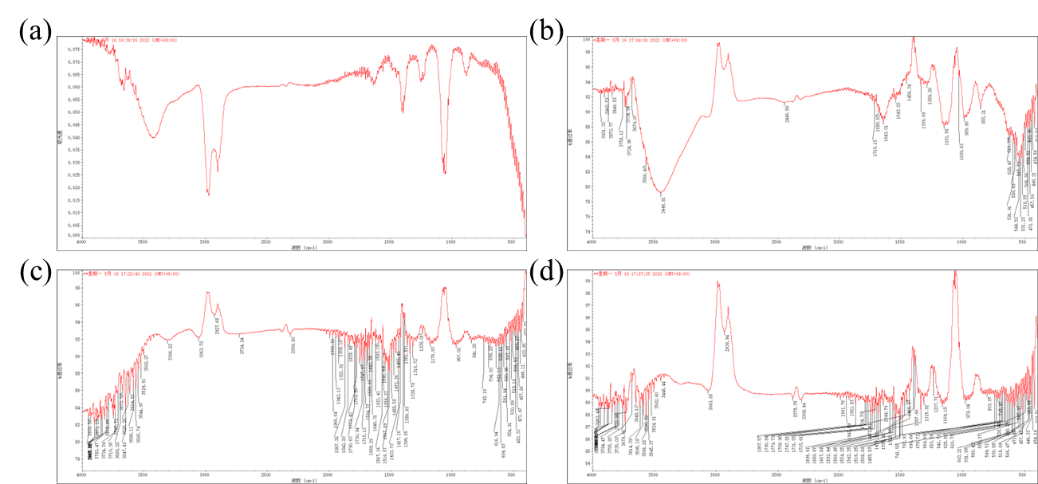


**Fig. S10.** **IR spectrum of (a) MBs, (b) activated MBs, (c) compound 5 and MBs, (d) MBs-Apt_63_.**
